# Diagnostic utility of CT in differentiating between ruptured ovarian corpus luteal cyst and ruptured ectopic pregnancy with hemorrhage

**DOI:** 10.1186/s13048-017-0374-8

**Published:** 2018-01-09

**Authors:** Xiaohong Liu, Litao Song, Jian Wang, Qin Liu, Yingna Liu, Xin Zhang

**Affiliations:** 1 Department of Radiology, Yueyang Hospital of Integrated Traditional Chinese and Western Medicine, Shanghai University of TCM, Shanghai, 200437 China; 2 The Second Military Medical University, Shanghai, 200433 China; 3 Department of Radiology, Seventh People’s Hospital affiliated with Shanghai University of TCM, Shanghai, 200137 China; 4 Department of Radiology, Changhai Hospital, Shanghai, 200433 China; 5 Department of Gynecology, Seventh People’s Hospital affiliated with Shanghai University of TCM, Shanghai, 200137 China; 6 Department of Radiology, Zhengzhou Central Hospital affiliated with ZhengZhou University, Henan, 450000 China

**Keywords:** Ruptured ovarian corpus luteal cyst (ROCLC), Ruptured ectopic pregnancy with hemorrhage (REPWH), Computed tomography (CT), Cyst shadow, Pelvic effusion

## Abstract

**Background:**

To evaluate the performance of computed tomography (CT) as a diagnostic aid to differentiate between ruptured ovarian corpus luteal cyst (ROCLC) and ruptured ectopic pregnancy with hemorrhage (REPWH).

**Methods:**

A total of 36 patients treated at our hospitals for ROCLC and REPWH from June 2014 to August 2017 were included in this study. Based on the diagnosis, the study population was divided into ROCLC group (*n* = 21) and REPWH group (*n* = 15). CT scans were performed for all patients prior to treatment. The size of the cystic shadows and the depth of the pelvic effusion were analyzed and compared with independent sample Student’s *t* test and Fisher’s exact test.

**Results:**

Cystic shadows with maximum diameters ≥3.0 cm presented in 16 patients with ROCLC and 1 patient with REPWH, while 4 patients with ROCLC and 9 patients with REPWH exhibited cystic shadows with maximum diameters <3.0 cm. The mean diameters along the major and minor axes in the two groups were 3.76 ± 1.11 cm and 2.93 ± 0.98 cm, 1.96 ± 0.65 cm and 1.60 ± 0.55 cm, respectively (*p* < 0.001*)*. The mean depth of the pelvic effusion in patients with ROCLC and REPWH were 5.20 ± 2.47 cm and 6.96 ± 2.07 cm, respectively (*p* = 0.038).

**Conclusion:**

The cystic shadow of ROCLC is larger than that of the REPWH. The depth of the pelvic effusion of REPWH is deeper than that of the ROCLC. CT can help differentiate between ROCLC and REPWH based on the size of the cystic shadow and the depth of pelvic effusion in the adnexal area.

## Background

Ruptured ovarian corpus luteal cyst (ROCLC) and ruptured ectopic pregnancy with hemorrhage (REPWH) are the most common gynecological emergencies in clinical settings [1–4]. However, ROCLC and REPWH are uncommon surgical acute abdominal symptoms. Thus, REPWH is often misdiagnosed as ROCLC, appendicitis, or ovarian cyst torsion owing to similar clinical presentation including acute onset of abdominal symptoms, persistent lower abdominal pain and other associated features of acute abdomen. Although the clinical presentation of ROCLC and REPWH is similar, the management approach is relatively different; most cases of ROCLC can be treated conservatively, whereas REPWH requires surgical intervention [5, 6]. Both ROCLC and REPWH account for a major proportion of mortality burden among healthy women of reproductive age. Over the past few years, incidence of gynecological acute abdomen in China has increased, especially after the relaxation of the two children policy in January 2016 (particularly the number of pregnant women above the average age has increased). Considering the likelihood of both clinical and radiologic misdiagnosis in women of child-bearing age who present with persistent pelvic pain and a large amount of pelvic fluid, it is vital for the radiologist to differentiate between ROCLC and REPWH. In recent years, reports of differentiating between ROCLC and REPWH using transvaginal ultrasonography and MRI have been increased [7–10]. However, fewer cases of ROCLC and REPWH were diagnosed using MRI in the clinical emergency settings. Additionally, MRI is not available in most emergency department of the hospitals, and it would take at least a few days even weeks to schedule a MRI evaluation; furthermore, ultrasonography is not available at some evening emergency rooms either. Therefore, CT is the only method that the clinicians would order if the patient were seen under such conditions. Traditionally, the diagnosis of ROCLC and REPWH is based on a combination of medical history, laboratory tests, and ultrasound findings. CT scan would not be the first-choice modality for diagnosing and differentiating ROCLC and REPWH due to the associated radiation hazard. However, with the increasing number of CT examinations in diagnosing gynecological acute abdomen, the use of CT to differentiate between ROCLC and REPWH may be justified, especially in emergency settings when patients have no clear medical history or in cases where ultrasound examination does not provide a definitive diagnosis. Amongst the recent articles on diagnosis and treatment of ROCLC and REPWH, few articles have reported the significance of CT for the differential diagnosis of ROCLC and REPWH. In a study by Liu et al. [11], abdominal CT imaging was found to be superior to ultrasound examination for achieving a definitive diagnosis of ROCLC. In the present study, the object was to evaluate the utility of CT in differentiating between ROCLC and REPWH based on the analysis of clinical records including imaging data and pathological results. The insights gained from this study may aid radiologists and clinicians in differentiating between these two conditions.

## Methods

This study was approved by our Institutional Review Board and Ethics Committee. Written informed consent was obtained from all patients prior to their enrollment.

### Study population

A total of 36 patients hospitalized with persistent abdominal pain treated at the Shanghai Seventh Hospital, Changhai Hospital, and Zhengzhou University affiliated Zhengzhou Central Hospital in the period between June 2014 and August 2017, were recruited. Of these, 21 patients (mean age, 30.71 ± 6.83 years; range, 16–42) were diagnosed with ROCLC (ROCLC group), while 15 patients (mean age, 32.73 ± 4.30 years; range, 27–41) were diagnosed with REPWH (REPWH group).

The diagnosis of ROCLC was confirmed by surgery in 3 patients and by clinical evaluation in 18 patients. The cause of abdominal pain in ROCLC group included intercourse (10 patients), external trauma (2 patients), bowel-movement (1 patient), and strenuous physical activity (1 patient). The remaining patients in the ROCLC group had no other obvious cause of abdominal pain, and one patient had no sexual history. All patients in the ROCLC group tested negative for HCG, and 9 patients had positive culdocentesis.

The diagnosis of REPWH was confirmed by surgery in 14 patients, while one patient was cured by conservative treatment. All patients in the REPWH group showed positive HCG; 4 patients had an in situ intrauterine device; 12 patients had a history of amenorrhea; 11 patients had a history of irregular vaginal bleeding; 4 patients presented positive culdocentesis. In REPWH group, patients’ gestational weeks were between 4 to 61 days, most of the gestational stage were less than 50 days (4 patients > 40 days), and one patient was 61 days.

### Clinical methods

All 36 patients in this study underwent abdominal CT using a 64-slice spiral CT scanner (Light Speed ​​VCT, GE Healthcare, Milwaukee, WI, USA) or the 2nd generation-dual-source Siemens CT scanner (SOMATOM Definition Flash, GER). Plain spiral CT scan was used, and no iodine contrast agents were administered during CT examination. Images were obtained from the anterior-superior iliac spine to the symphysis pubis or from the diaphragmatic dome to the symphysis pubis. The detailed parameters were as follows: tube voltage,100~130 KV and 200~250 Ma; pitch factor, 1.0; layer thickness, 5 mm; layer space, 5 mm; matrix, 512; delthyrial cavity, 1.00 mm, and the thinner reconstructed section interval, 1.2 mm.

### CT image analysis

**Fig. 1:**
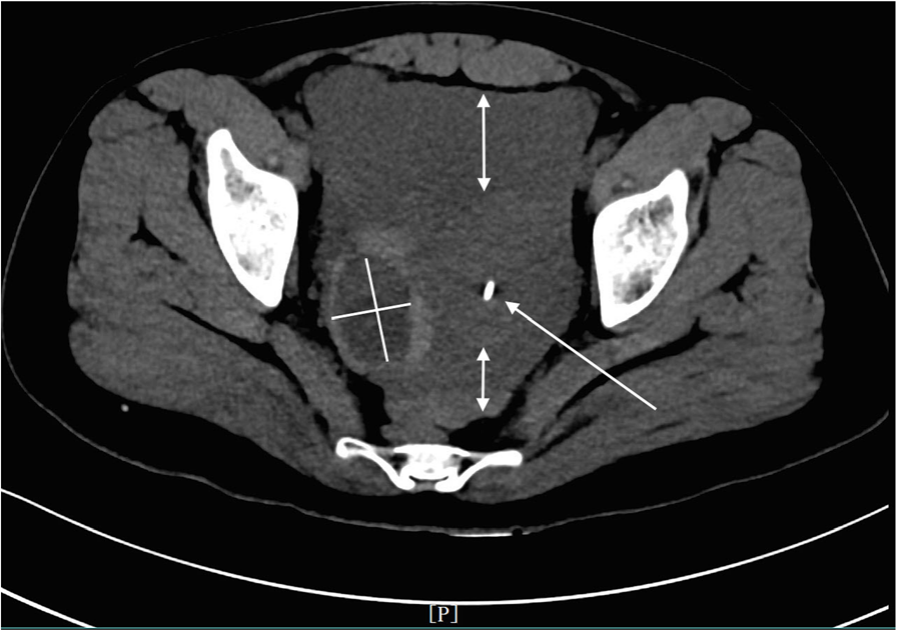
CT findings of ROCLC. CT image of a post-coital patient who presented with persistent abdominal pain for a period of half a day; HCG test was negative. The crosshair indicates the maximum and minimum diameter of the ruptured corpus luteal cyst; the double-headed arrows indicate the depth of pelvic fluid; the long arrow indicates the location of the uterus and IUD

Images were reviewed and analyzed by radiologists who had more than 5 years of experience in imaging diagnosis. The cystic shadow on the side of uterus was measured by taking the maximum diameter along the major axis of the cystic shadow (Fig. 1); pelvic effusion was measured from the deepest surface of the uterus to the front of the rectum and from the front of the uterus to the front of the abdominal wall (Fig. 1). In this study, metric measurement units were utilized (“centimeter” with accuracy to the first decimal place).

### Statistical analysis

The data was analyzed with SPSS software (Version 22.0, Chicago, IL, USA). The diameter of the cystic shadows and the depth of pelvic effusion were compared using the independent sample Student’s *t* test and Fisher’s exact test. All variables analyzed by *t* test were assessed for normal distribution. For the purpose of this analysis, ovarian corpus luteum with a diameter greater than 3 cm was defined as a corpus luteal cyst [3]. Between-group differences associated with a *p* value <0.05 were considered statistically significant.

## Results

### Clinical and surgical parameters

All patients included in this study had undergone CT examination prior to treatment. In the ROCLC group, ruptured sites and corpus luteal cysts were noticed after opening up the blood clots during surgery. Active bleeding was observed at the site of rupture; the volume of the pelvic fluid ranged from 300 mL–3000 mL. In the REPWH group, 14 cases were confirmed by surgery, whereas one patient was cured by conservative treatment. Twelve REPWH patients experienced ruptured tubal pregnancy; the location of the tubal pregnancy was ampulla portion (6 patients), isthmus portion (3 patients), intramural area (2 patients), and the fimbria area (1 patient). Fallopian tubes were found thickened during surgery; the surface of the fallopian tube was purple and blue, and active bleeding was seen at the ruptured site. Two patients experienced ruptured ovarian ectopic pregnancy. Among patients with ovarian pregnancy, purple and blue bulging presented on the surface of the ruptured site; small blood clots along with severe hemorrhage were noticed in the ovaries. Severe hemorrhage and large blood clots were noticed in the Douglas pouch of the peritoneal cavity (Table 1).

**Table 1:** Clinical and surgical parameters of ROCLC and REPWH

	ROCLC	REPWH
Total patients	21	15
Age(year)	30.71 ± 6.83	32.73 ± 4.30
Surgery (n)	3	14
Cystic shadow ≥3.0 cm (n)	16	1
Cystic shadow	4	9
<3.0 cm (n)		
Pelvic fluid	21	15

Note: Diameter along the major axis of the cystic shadows was analyzed using SPSS software package; between-group differences were assessed with Fisher’s exact test. More patients in the ROCLC group exhibited a cystic shadow ≥3 cm in diameter along the major axis as compared to that in the REPWH group

### CT image findings of ROCLC and REPWH

**Fig. 2:**
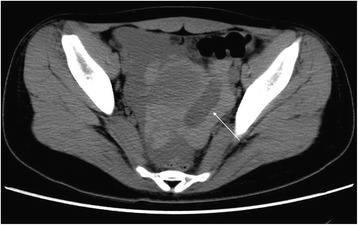
CT findings of ROCLC. CT image of a post-coital patient who presented with persistent abdominal pain for a period of half a day; HCG test was negative. Long single arrow indicates a homogenous low-density cystic shadow

**Fig. 3:**
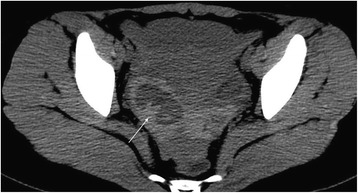
CT findings of ROCLC. CT image of a post-coital patient who presented with persistent abdominal pain for a period of 1 day and negative HCG test. The long arrow indicates the serrated shape of the ruptured ovarian corpus luteal cyst

**Fig. 4:**
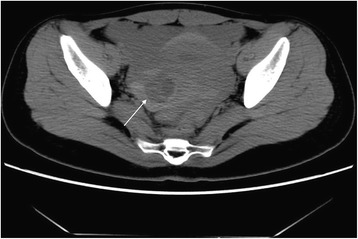
CT findings of ROCLC. CT image of a post-coital patient who presented with persistent abdominal pain for a period of 1 day and negative HCG test. Single arrow indicates mixed-density blood clots inside the cystic shadow

**Fig. 5:**
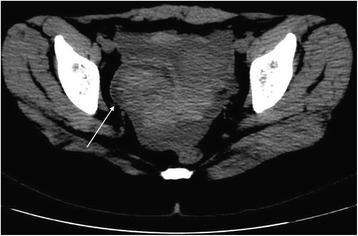
CT findings of ROCLC. CT image of a patient who presented with persistent abdominal pain for a period of 2 hours with no clear cause; HCG test was negative. Culdocentesis revealed signs of active bleeding. Single arrow indicates the ruptured corpus luteal cyst filled with high-density blood

**Fig. 6:**
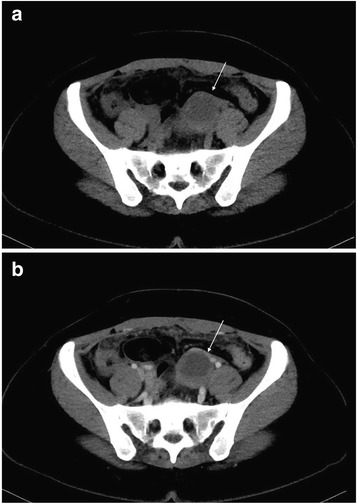
**a** and **b**. CT radiographs of the same patient with plain and enhanced imaging. Single arrow in Fig. 6**a** indicates left ruptured ovarian corpus luteal cyst; single arrow in Fig. 6**b** indicates the clearer image of the left ruptured ovarian corpus luteal cyst on enhanced CT scan

**Fig. 7:**
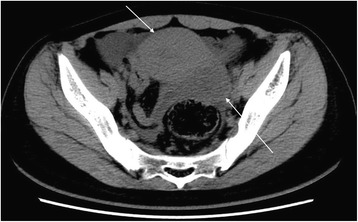
CT findings of ROCLC. The patient presented with persistent abdominal pain for a period of half a day with no clear cause; HCG test was negative. The longer arrow indicates the lack of any obvious cystic shadow in the left ruptured ovarian luteal cyst; the shorter arrow indicates the location of the uterus

**Fig. 8:**
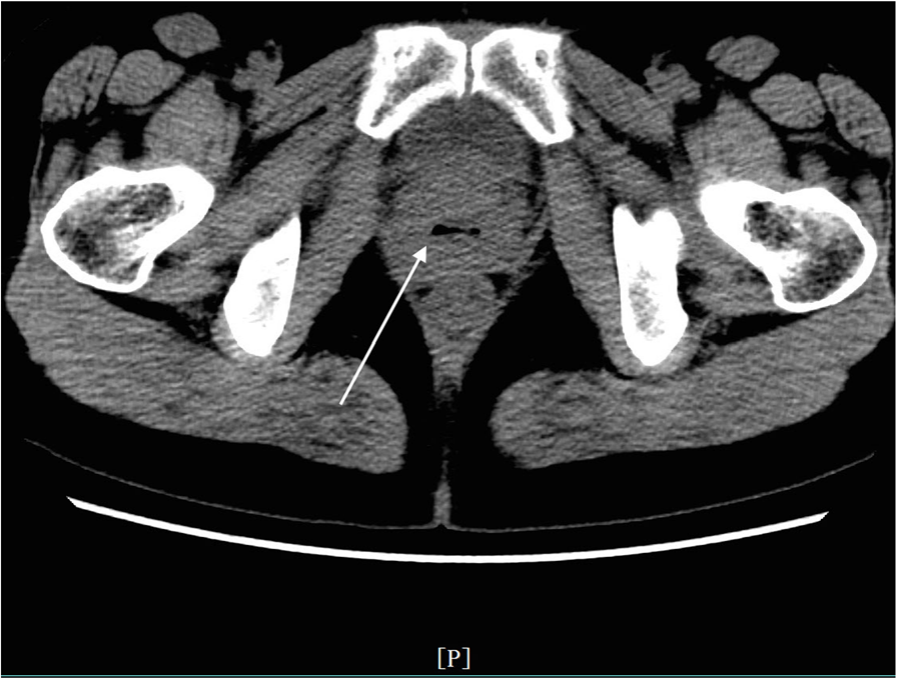
CT findings of ROCLC. Patient presented with persistent abdominal pain for a period of 2 hours which started after intercourse; HCG test was negative and there were no signs of pelvic hemorrhage. The single arrow indicates the air shadow in the cervix

The maximum and minimum diameters of the cystic shadow and the depth of the pelvic effusion were compared between the two groups (Table 2). Cystic shadows were found in all 21 patients in the ROCLC group, which mimicked the “Ring” sign on CT imaging. 1) The density of the cystic shadow was varied: mixed- density cystic shadow in the adnexal area was observed in 20 patients. Of these, 9 patients exhibited low density in the central area and high density on the edge of the ring area; 7 patients exhibited mixed-density in the central area and high density on the edges of the ring area; 4 patients exhibited high density in the central area and on the edges of the ring area. In 1 patient, no obvious cystic shadow was observed in the adnexa. 2) Size and location of the cystic shadows: cystic shadows were noticed on the right side of the adnexal area in 13 patients and on the left side in 7 patients. The maximum diameter of the cystic shadow was 6.3 cm, while the minimum diameter was 2.0 cm. 3) CT attenuation values of the cyst edge and the pelvic fluid were 31 HU~91 HU and 22 HU~105 HU, respectively. 4) Pelvic fluid depth: 1.3 cm~8.3 cm. 5) Summary of CT findings and figure illustrations of the ROCLC group: Twenty patients in the ROCLC group exhibited large cystic shadow of heterogenous density in the adnexal area. The cystic shadow was ring-shaped, largely smooth-walled and was surrounded by mixed-density blood clots (Fig. 2); CT showed significant discontinuous rupture sites in the wall of some cysts (Fig. 3); mixed-density blood clots were found inside some of the cystic shadows (Fig. 4); high-density blood clots were observed in some of the cystic shadows, while low-density blood clots were observed at the edges; mixed- density blood clots were observed around most of the outer edges (Fig. 5); enhanced posterior wall showed incomplete strengthening on the ring area (Fig. 6a, b); mixed amorphous density bleeding in the adnexal area without cystic shadow were noticed in a small number of patients; the depth of effusion in the pelvic area tended to vary (range, 300 mL~3000 mL) (Fig. 7); accumulated air was observed at the cervical area in the post-coital patients (Fig. 8).

**Table 2:** Comparisons of the diameter of the cystic shadows and the depth of the pelvic effusion between the ruptured ovarian corpus luteum cyst and ruptured ectopic pregnancy with hemorrhage $\left(\overline{\mathrm{x}}\kern0.5em \pm \kern0.5em \mathrm{s}\right)$

Groups	No.of patients	Maximum diameter of the cystic shadows (cm)	Minimum diameter of the cystic shadows (cm)	No. of patients	The depth of the pelvic effusion (cm)
ROCLC	20	3.76 ± 1.11	2.93 ± 0.98	21	5.20 ± 2.47
REPWH	10	1.96 ± 0.65	1.60 ± 0.55	15	6.96 ± 2.07
*t* value	–	4.7	3.96	–	−2.16
*p* value	–	0.000	0.000	–	0.038

**Fig. 9:**
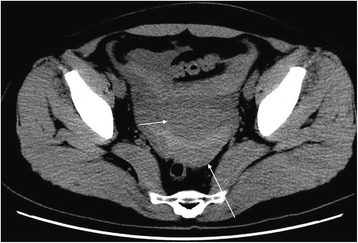
CT findings of REPWH. The patient presented with lower abdominal pain for a period of 2 h. This patient had a history amenorrhea for 2 months and irregular vaginal bleeding for 1 month; β-HCG test was positive; no signs of gestational sac were observed; left isthmus ectopic pregnancy was confirmed by surgery. The single arrow indicates the mixed-density pelvic effusion; the front shorter arrow indicates the location of the uterus, which does not exhibit any signs of cystic shadow

**Figs. 10:**
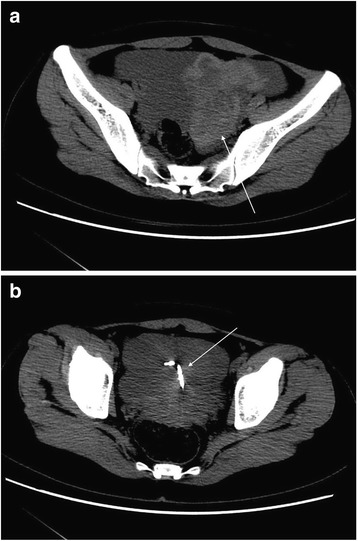
**a** and **b**. CT findings of REPWH for the same patient. Patient experienced persistent abdominal pain with no clear cause; HCG test was weakly positive. Left ovarian ectopic pregnancy rupture was confirmed by surgery. The single arrow indicates the mixed-density blood and IUD in the left adnexal area

**Fig. 11:**
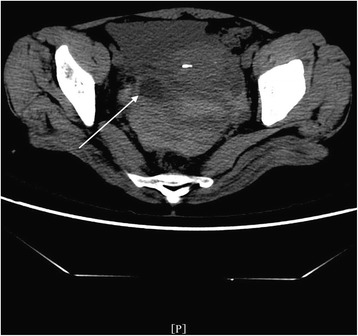
CT findings of REPWH. The patient had a history of amenorrhea for 54 days and a positive HCG test. Right ovarian ectopic pregnancy rupture was confirmed by surgery. The single arrow indicates a cystic shadow (18 mm × 19 mm) on the right side of the adnexal area

In the REPWH group, 14 patients were confirmed by surgery and 1 patient was confirmed by clinical pathology. Of the 14 surgically treated patients, 12 patients had tubal pregnancies while 2 had ovarian pregnancy. 1) CT findings of tubal pregnancy: Of the 12 patients with tubal pregnancy, cystic shadow in the adnexal hemorrhagic area with mix-density blood clots were observed in 8 patients, while mixed-density adnexal hemorrhage was found in the other 4 patients with no obvious cystic shadows. Cystic shadow was found in the right adnexal area in the sole patient who was treated conservatively. The deepest pelvic effusion was 10.3 cm. 2) CT findings in patients with ovarian pregnancy: Out of the two patients with ovarian pregnancy, 1 patient did not exhibit any obvious cystic shadow in the hemorrhagic area, while the other patient exhibited a 1.8 cm × 1.9 cm cystic shadow of mixed-density in the hemorrhagic area. The deepest pelvic effusion was 8.6 cm. 3) Size and location of the cystic shadow: In 6 patients, the shadows were on the right side of the adnexal area, while in 4 patients, the shadows were on the left side. The maximum and minimum diameters along the major and minor axes of the cystic shadow were 3.0 cm and 0.7 cm, respectively; 4) CT attenuation values of the inner area and at the edge of the cystic shadow were 17 HU~64 HU and 25 HU~73 HU, respectively; 5) The depth of the pelvic effusion ranged from 3.7 cm~10.3 cm; 6) CT attenuation value of the pelvic effusion: 22 HU~82 HU; 7) Summary of the CT findings and figure illustrations of the REPWH group: mixed density amorphous bleeding was observed in the pelvic adnexal area (Fig. 9). In some patients, mixed density blood clots and IUD were observed in the uterus or in the adnexal area (Fig. 10a, b); in some patients, a smaller cystic shadow was found in the hemorrhagic area (Fig. 11). Accumulated mixed-density blood clots were observed in the pelvic area (The density of the blood clots mainly correlated to the time of bleeding). Size of uterus was normal or slightly enlarged; however, the uterine enlargement was smaller than that observed in a normal pregnant uterus.

## Discussion

The main findings of this study are as follows: cystic shadows and pelvic effusion were the key findings observed on CT images. The mean maximum diameter of the cystic shadow in the ROCLC and REPWH groups was 3.76 ± 1.11 cm and 1.96 ± 0.65 cm, respectively; the mean depth of the pelvic effusion was 5.20 ± 2.47 cm and 6.96 ± 2.07 cm, respectively. Owing to the hemorrhagic content, cystic shadows presented as different density shadows on CT images. Depending on the duration and amount of the hemorrhage, they could be in uniformed liquid density, or a mixed of high and low density, or slightly higher density in most area. There are always certain edges on the outside of the cystic shadows, and the shape of the edges could be continuous or discontinuous. When there was no cystic shadow shown in the adnexal area, the hemorrhage on the side of the adnexa appears to be a mixed-density shadow with irregularly shaped and blurred edges, and the adjacent peritoneal is blurred and thickened. The density of the pelvic effusion showing on the CT image could be at the same attenuation, or mixed, or slightly higher density in most of the area, and the density is mainly correlated to the state of the hemorrhage coagulation. Therefore, the density of the pelvic effusion is higher in the coagulation area.

Ovarian corpus luteum is formed post ovulation; the growth of the ovarian corpus luteum is associated with an increase in luteal blood flow and increased serum progesterone levels [3, 4]. The size and function of the corpus luteum is at its peak 7 days after ovulation (diameter: 1–2 cm) [3, 4]. It is designated as a corpus luteal cyst when the diameter of the corpus luteum is >3 cm [3, 4]. Most ROCLC occur in women of reproductive age-group who have hyper-functional ovaries [5]. ROCLC may be caused by sexual activity, gynecological examination or any other external impact; it may also rupture spontaneously, such as during bowel-movement. Ovarian luteal cyst rupture tends to occur during the later phase of the menstruation and after sexual intercourse [11, 12]. The characteristics of ROCLC are as follows: No history of amenorrhea, vaginal bleeding, early pregnancy signs, acute onset, sudden pain at one side of the lower abdomen, which gradually spreads to the entire abdomen, and negative HCG test. ROCLC is a self-limiting disease, as most of the ruptured sites tend to undergo self-healing. Chances of recurrent bleeding in ROCLC are relatively low, because it can self-agglutinate leading to cessation of bleeding after one bleeding episode. Thus, most of the ROCLC patients do not require surgical treatment [9, 12, 13].

Ectopic pregnancy or extrauterine pregnancy refers to the implantation of the fertilized ovum outside of the uterus. The condition is a major threat to the health of women in the child-bearing age group [6]. It is one of the major complications encountered during the first trimester of pregnancy, and it has been reported that 1.3–2.4% of all pregnancies are ectopic pregnancies [6]. Most ectopic pregnancies occur in women in the age-group of 30–40 years and tubal pregnancy is the most common type of ectopic pregnancy [8, 14–17]. Ectopic pregnancy may occur in the ovaries, abdominal cavity, broad ligament, and cervix; however, these are rare sites of ectopic pregnancy [7, 18]. The main clinical symptoms of ectopic pregnancy include history of amenorrhea, abdominal pain, vaginal bleeding, and positive β-HCG. The diagnosis can be confirmed based on the medical history and positive culdocentesis; however, the diagnosis may not be straightforward in patients with negative culdocentesis, especially in those with a history of irregular menstrual periods. Ectopic pregnancy is very likely to be misdiagnosed in patients with no typical clinical symptoms if only traditional urine tests are assessed.

However, on CT imaging, mixed density blood clots, “Ring” sign of cystic shadow and pelvic fluid can be observed. In cases of ROCLC, large cystic shadows can be seen in the hemorrhage area. The diameters of the cystic shadows are generally >3.0 cm. The mean depth of pelvic effusion in this study was 5.20 ± 2.47 cm. In the REPWH group, cystic shadows were observed on CT images, as well. The maximum diameter along the major axis of cystic shadow in REPWH group was generally <3.0 cm and the mean depth of the pelvic fluid in this study was 6.96 ± 2.07 cm. Therefore, the size of the cystic shadow associated with ROCLC is larger than that of the REPWH and the depth of pelvic effusion in patients with REPWH is greater than that in patients with ROCLC.

In order to diagnose and differentiate REPWH and ROCLC in emergency settings, it is also necessary to exclude the possibility of ovarian cyst torsion and other causes of acute abdomen due to the similar clinical presentation. In elderly patients, acute abdominal pain could be due to sudden changes in body position or posture, such as a sudden stop when travelling on a bus. Some patients may have a preexisting ovarian cyst or tumor, and ovarian cyst torsion could occur at any age; hence, an accurate differential diagnosis is vital.

In recent years, magnetic resonance imaging (MRI) has been increasingly employed for the diagnosis of acute gynecological conditions with abdominal symptoms [19]. However, most hospitals do not have an MRI available at the emergency department. Ultrasound is unable to provide a clear diagnosis in approximately 25% of all cases who present with acute abdomen compatible with gynecological conditions. Additionally, some hospitals do not have ultrasonography available at night, either. When CT was not commonly used, ROCLC was frequently misdiagnosed preoperatively as appendicitis, ectopic pregnancy, endometriosis, or neoplasm [11, 12, 19]. In emergency settings, prompt diagnosis is of critical importance in cases who present with acute gynecological abdominal symptoms with no clear medical history, especially, when ultrasound is unable to provide a clear diagnosis. In such cases, CT examination may confer a distinct advantage as it can not only detect cystic solid masses in the pelvis, but can also delineate pedicle torsion with careful observation [19, 20]. Lee et al. [10] reported that the diagnosis of ROCLC is confirmed if active bleeding is present and a certain amount of pelvic effusion is shown on the CT scan. Therefore, CT scan is invaluable in the differential diagnosis of acute abdomen and offers a distinct value addition in differentiating between ROCLC and REPWH. In addition, CT scan can clearly delineate the site of bleeding and help assess the amount of bleeding. On CT imaging, we quantified the amount of effusion as the depth of pelvic fluid is greater than 30 HU in CT attenuation [5]. CT can also accurately determine the presence of active pelvic bleeding. After the exclusion of general causes of acute abdominal symptoms, such as appendicitis, ureteral stones, and other diseases, ROCLC can be confirmed if there is obvious bleeding in the pelvic area and the major axis of the mixed-density bleeding mass at one side of the uterus or adnexal area is greater than 3.0 cm, especially in patients who are at the middle or towards the end of their menstrual cycle and if same symptoms were present during intercourse prior to the start of abdominal pain. REPWH can be confirmed if there is extensive hemorrhage in the pelvis, or the major axis of cystic shadow in the mixed -density hemorrhagic area on one side of the uterus or in the adnexal area is less than 3.0 cm in size; additionally, these patients have a history of amenorrhea and positive β-HCG. In the REPWH group, there were 2 patients with right fallopian tube ectopic pregnancy with a left ovarian cyst greater than 3.0 cm. Identification of the site of bleeding in conjunction with medical history and results of HCG test can help distinguish ROCLC from REPWH. We also need to correlate the side of bleeding and the site of abdominal pain along with the assessment of the peri-cystic structures. Thus, CT scan can aid in the differential diagnosis of ROCLC and REPWH when ultrasound is unavailable or is unable to provide clear diagnosis in emergency situations. There is some overlap between the CT findings of ROCLC and REPWH, such as when the major axis of the cystic shadow in the adnexal bleeding area is approximately 3.0 cm, or when no cystic shadow is present. In the current study, the proportion of patients who did not exhibit any cystic shadow was higher in the REPWH group as compared to that in the ROCLC group, which is consistent with the findings reported by Bi et al. [20] Under such circumstances, the results of HCG test and the patient’s menstrual cycle status can also help improve the accuracy of diagnosis based on CT findings.

The limitations of this study include the relatively small sample size of patients, and inclusion of only 2 patients with ovarian ectopic pregnancy. Most of the cases were tubal ectopic pregnancy, and other types of ectopic pregnancy were not available for inclusion. Therefore, a prospective, randomized, multicenter study of ectopic cases should be conducted to validate our findings.

## Conclusion

In conclusion, CT imaging can be invaluable for differentiating between ROCLC and REPWH by observing the size and presence of the cystic shadow and depth of the pelvic effusion in the adnexal area.

## Abbreviations

REPWH: Ruptured ectopic pregnancy with hemorrhage
ROCLC: Ruptured ovarian corpus luteal cyst

## References

[1] Li N-N, Duan X-H, Liu B (2012). CT diagnosis of common gynecological acute abdomen [J]. Journal of practical. Radiology.

[2] GD MW, Hill MJ, Dietrich CS (2008). Gynecologic emergencies. Surg Clin North Am.

[3] Hertzberg BS, Kliewer MA, Paulson EK (1999). Ovarian cyst rupture causing hemoperitoneum: imaging features and the potential for misdiagnosis. Abdom Imaging.

[4] Baerwald AR, Adams GP, Pierson RA (2005). Form and function of the corpus luteum during the human menstrual cycle. Ultrasound Obstet Gynecol.

[5] Kim JH, Lee SM, Lee JH, Jo YR, Moon MH, Shin J (2014). Successful conservative management of ruptured ovarian cysts with hemoperitoneum in fair women [J]. PLoS One.

[6] Taran F-A, Kagan K-O, Hübner M, Hoopmann M, Wallwiener D, Brucker S (2015). The diagnosis and treatment of ectopic pregnancy[J]. Dtsch Arztebl Int.

[7] Roche O, Chavan N, Aquilina J, Rockall A (2012). Radiological appearances of gynaecological emergencies. Insights into Imaging[J].

[8] Kao LY, Scheinfeld MH, Chernyak V, Rozenblit AM, Oh S, Dym RJ (2014). Beyond ultrasound: CT and MRI of ectopic pregnancy[J]. AJR Am J Roentgenol.

[9] Mohamed M, Al-Ramahi G, McCann M (2015). Postcoital hemoperitoneum caused by ruptured corpus luteal cyst: a hidden etiology[J]. J Surg Case Rep.

[10] Lee MS, Moon MH, Woo H, Sung CK, Jeon HW, Lee TS (2017). Ruptured corpus Luteal cyst: prediction of clinical outcomes with CT[J]. Korean J Radiol.

[11] Liu Q-H, Liu X-Z (2016). Ultrasonography, CT findings and diagnostic value of rupture and hemorrhage of ovarian Anthurocysts [J]. Chinese Journal of CT and MRI.

[12] Jin C-Z, Yao L-X, Tan W-C (2012). CT diagnosis of ruptured ovarian corpus luteum [J]. Journal of Practical Radiology.

[13] Choi HJ, Kim SH, Kim SH, Kim HC, Park CM, Lee HJ (2003). Ruptured corpus luteal cyst: CT findings[J]. Korean J Radiol.

[14] Kirsch JD, Scoutt LM (2010). Imaging of ectopic pregnancy. Applied Radiology[J].

[15] Marion LL, Meeks GR (2012). Ectopic pregnancy: history, incidence, epidemiology, and risk factors [J]. Clin Obstet Gynecol.

[16] Chen L, Qiu L, Diao X, Yue Q, Gong Q (2015). CT findings of omental pregnancy: a case report. Jpn J Radiol[J].

[17] Cai Y-Y, Xiao E-H, Shang Q-L, Xiao L-Z (2017). Ectopic pregnancy in the liver incidentally diagnosed by imaging: a case report[J]. Experimental and Therapeutic Medicine.

[18] Parker RA, Yano M, Tai AW (2012). MR imaging findings of ectopic pregnancy: a pictorial review[J]. Radiographics.

[19] Cano Alonso R, Borruel Nacenta S, Díez Martínez P (2009). Role of multidetector CT in the management of acute female pelvic disease [J]. Emerg Radiol.

[20] Bi X-J, Zhang Q, Zhang X-Q (2016). CT and MRI findings of ectopic pregnancy [J]. Journal of Clinical Radiology.

